# Citizen Responses to Government Restrictions in Switzerland During the COVID-19 Pandemic: Cross-Sectional Survey

**DOI:** 10.2196/20871

**Published:** 2020-12-03

**Authors:** Kevin Selby, Marie-Anne Durand, Alexandre Gouveia, Francesca Bosisio, Gaia Barazzetti, Maxime Hostettler, Valérie D'Acremont, Alain Kaufmann, Christian von Plessen

**Affiliations:** 1 Center for Primary Care and Public Health (Unisanté) University of Lausanne Lausanne Switzerland; 2 University Toulouse III Paul Sabatier Toulouse France; 3 The ColLaboratory - Participatory, Collaboratory and Action-Research Unit University of Lausanne Lausanne Switzerland; 4 Institute for Clinical Research University of Southern Denmark Odense Denmark

**Keywords:** COVID-19, coronavirus, Switzerland, mitigation strategies, citizen knowledge

## Abstract

**Background:**

The success of government-recommended mitigation measures during the COVID-19 pandemic depends largely on information uptake and implementation by individual citizens.

**Objective:**

Our aim was to assess citizens’ knowledge and perceptions about COVID-19 recommendations in the Canton of Vaud, Switzerland.

**Methods:**

A cross-sectional electronic survey with open and closed questions was disseminated by community-based partners prior to the relaxation of government restrictions. Outcomes included citizen knowledge (9-question measure) and worry about the virus, perception of government measures, and recommendations for improvements. Comparisons used linear regression, controlling for age, sex, education, and health literacy. Free-text answers were analyzed thematically.

**Results:**

Of 807 people who accessed the survey, 684 (85%) completed all questions and 479 (60%) gave free-text recommendations. Overall, 75% were female, the mean age was 48 years, and 93% had high health literacy. Knowledge scores were high, with a median score of 8 out of 9. Mean levels of worry about the COVID-19 pandemic were higher in women than men (55/100 versus 44/100, *P*<.001), and in respondents with lower health literacy (57/100 versus 52/100, *P*=.03). Self-reported adherence to recommendations was high (85%) and increased with age and worry (both *P*<.001). Respondents rated their own adherence higher than others (85% versus 61%, *P*<.001). Moreover, 34% of respondents reported having self-quarantined; this rose to 52% for those aged ≥75 years. Those who had self-quarantined reported higher levels of fear. Nearly half (49%) of respondents felt the government response had been adequate, though younger age and higher levels of worry were associated with considering the response to be insufficient (both *P*<.001). Analysis of open-text answers revealed 4 major themes: access to and use of masks, gloves, and hand sanitizer; government messaging; lockdown and lockdown exit plan communication; and testing for COVID-19.

**Conclusions:**

Knowledge, adherence, and satisfaction regarding government recommendations and response were high in this sample, but many desired greater access to personal protective equipment. Those with lower health literacy and those who have been in self-isolation reported greater concerns about the pandemic.

## Introduction

The global threat posed by the COVID-19 pandemic has led governments to impose unprecedented restrictions on personal movement and conduct [1]. On February 25, 2020, Switzerland had its first confirmed case; just 4 weeks later, there were 10,772 cases and 165 deaths [2]. On March 13 and 16, federal public health officials announced restrictions on gatherings and recommended multiple behavior changes intended to slow the spread of the virus and protect those most vulnerable to severe outcomes of infection [3,4]. Further, testing for COVID-19 was limited to high-risk populations. These restrictions depended largely on individuals self-isolating in case of symptoms and self-quarantining in case of contact with someone with symptoms. The government relied on cooperation from the public to adapt policies to their own situation. Little is known about how individuals understood, perceived, and implemented the recommendations in their personal life. Adherence to recommendations is typically influenced by the individual’s understanding of the information, perception of the virus, related threat, and perceived impact on their lives. Rapid online surveys of people’s perceptions during the COVID-19 outbreak suggested that participants generally had a good knowledge of the primary disease transmission modes and common symptoms [5]. However, about one-third of the sample had misconceptions about ways to prevent an infection and recommended care-seeking behaviors. In India, a cross-sectional online survey of 2459 participants also revealed good knowledge, which was higher among respondents aged >40 years, people with higher educational attainment, those living in urban areas, and those in health care professions [6]. As far as could be determined in April 2020, no other survey of adult knowledge and perceptions of the COVID-19 pandemic had been conducted in Switzerland.

In this context, we aimed to assess citizens’ knowledge and perceptions about COVID-19 recommendations in the Canton of Vaud, Switzerland. Further, we collected information about individual behaviors and perceived adherence to population-level recommendations and suggestions for improvement.

## Methods

We conducted a cross-sectional electronic survey using REDCap (Vanderbilt University) between April 17 and 28, 2020, prior to the easing of restrictions in Switzerland. The survey was distributed on social media by multiple community partners, including a local consumer organization, health promotion organizations, and university community members. Ethics approval was not required as all data collected were anonymous [7]. The survey was supported by the health department of the Canton of Vaud, a French-speaking canton in Switzerland.

The electronic survey consisted of 20 questions about participants’ knowledge and perception, as well as individual behaviors and perceived adherence, regarding population-level recommendations about COVID-19 (Multimedia Appendix 1). The survey also assessed information sources and worry. We used an open-text response to collect suggestions for improvement regarding additional measures that the government could implement to limit the spread of the virus (see Table 1 for an overview of the survey structure). The survey was developed in English, translated to French, and user-tested by 6 non–medical professionals. Demographic data collected included age, sex, number of persons in household, Canton of residence, highest level of education, a validated health literacy item [8], and whether they had been tested for COVID-19. A knowledge score was developed with 9 true-false items about current government recommendations. We used lists of common changes due to the restrictions, enabling factors and barriers to implementation, and protection measures. Visual analogue scores were used to measure worry about the pandemic, self-reported adherence, and perception of government measures.

**Table 1:** Overview of survey structure.

Question number	Section
1-6	Sociodemographic questions
7-8	COVID-19 testing and symptoms
9	Information sources about COVID-19
10	Worry about the new coronavirus
11	Knowledge about government restrictions
12-18	Individual behaviors and perceived adherence to recommendations
19	Perception of the government measures to limit the spread of the new coronavirus
20	Additional suggestions to limit the spread of the new coronavirus (open-text answer)

We used descriptive statistics for participant characteristics, including means for continuous variables, except for perceptions of government measures, which were dichotomized because of a nonnormal distribution. Scores below 45 were classified as considering the government response inadequate. We used multivariable regression to explore associations between participant characteristics and level of worry and self-reported adherence; both models included participant age (continuous), gender (male or female), education (university level or not), health literacy (high or low), and knowledge of current government recommendations (number of answers correct). We used logistic regression to explore associations between considering government measures inadequate and participant age, gender, education, health literacy, knowledge of current government recommendations, and worry. The estimation command “predict” was used in STATA (StataCorp) to calculate absolute differences between groups, using the models controlling for other factors. Analyses were limited to completed surveys. A *P* value <.05 was considered statistically significant. All quantitative analyses were performed with STATA and free-text answers were analyzed thematically by one author (MD) using MAXQDA (VERBI Software).

## Results

### Overview

Of 807 people who accessed the survey, 684 (85%) completed it and 479 (60%) gave free-text recommendations for improvement. Respondents were 75% female, with a mean age of 48 years and 93% self-reporting high health literacy (Table 2). The primary sources of information about COVID-19 were television and radio (73%), government sources (72%), and newspapers (63%). Knowledge scores were high, with a median score of 8/9. The most common incorrect answer was agreeing that people must stop using all public transport, which was not a federal recommendation (32% incorrect). Overall, 34% of respondents reported having self-quarantined, a proportion that increased with age (27% of those aged >30 years versus 52% for those aged ≥75 years, *P*<.001).

**Table 2:** Demographic characteristics of respondents who completed the questionnaire (N=684).

Characteristic		Values, n (%)
**Age (years)**		
	18-39	223 (33)
	40-64	351 (51)
	≥65	110 (16)
**Sex**		
	Female	165 (24)
	Male	518 (76)
**Canton of residence (31 missing)**		
	Vaud	581 (88)
	Other	77 (12)
**Highest education level (5 missing)**		
	High school or apprenticeship	239 (35)
	University or professional school	440 (65)
**Health literacy^a^ (1 missing)**		
	Low health literacy	48 (7)
	High health literacy	635 (93)
**Tested for COVID-19 by reverse transcription polymerase chain reaction test (1 missing)**		
	Yes	44 (6)
	No	639 (94)
**Members in household (4 missing)**		
	≤2	365 (53)
	≥3	315 (47)
**Knowledge score on 9 true-false questions**		
	≤7	222 (33)
	8 or 9	461 (67)

^a^Dichotomized, with “Often” and “Always” as high health literacy, and “Never,” “Rarely,” and “Sometimes” as low health literacy.

**Figure 1:**
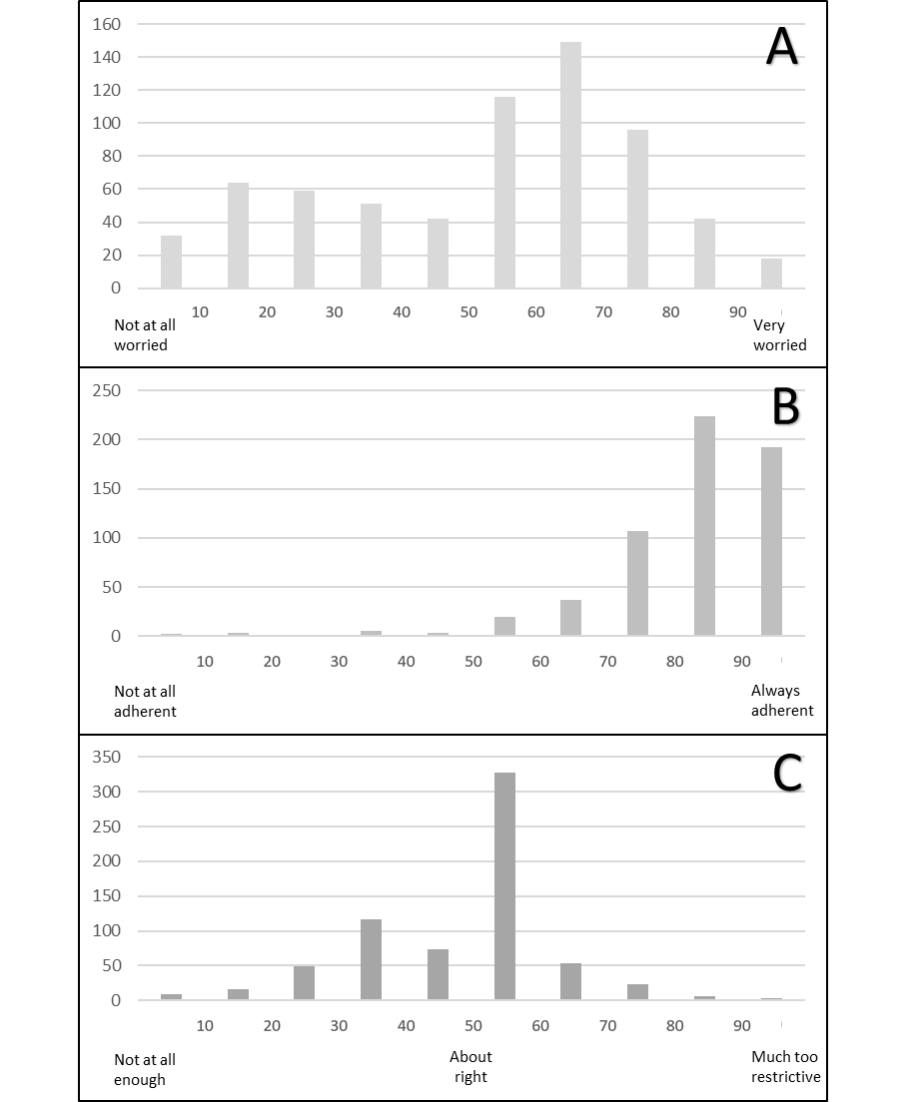
Distribution of participant responses to three questions using a visual analogue scale (0-100). (A) Overall worry about COVID-19. (B) Self-reported adherence to federal recommendations. (C) Adequacy of government recommendations to limit the spread of COVID-19.

After controlling for participant age, level of education, and knowledge of current government recommendations, mean levels of worry were higher in women than in men (55/100 versus 44/100, *P*<.001), in respondents with lower health literacy (57/100 versus 52/100, *P*=.03), and among those who had been in self-isolation or self-quarantine (59/100 versus 49/100, *P*<.001). The distribution of responses is shown in Figure 1. Nearly half (49%) of respondents considered the government response to have been adequate, responding in the middle of the 0-100 scale (Figure 1). Younger age and higher worry were associated with considering the response to be insufficient (both *P*<.001). Self-reported adherence to government recommendations was high (85%) and increased with age and level of worry (both *P*<.001; Figure 1). Self-reported adherence was higher than the perceived adherence of other people (85% versus 61%, *P*<.001). Government (82%) and other online sources of information (38%), help from family and friends (32%), and employers (25%) were considered to be useful for following the recommendations. Basic needs (57%), family obligations like caring for the elderly (20%), and difficulties with changing habits (10%) were barriers. The majority of respondents had intervened to encourage others to follow recommendations (77%).

### Thematic Analysis

Thematic analysis of open-text recommendations for improvements to the government response revealed 4 major themes, presented below alongside participant remarks.

#### Access and Use of Masks, Gloves, and Hand Sanitizer

Many felt masks should become compulsory outside the home, particularly in crowded areas (eg, public transport, at work), where social distancing is difficult.

We should implement the use of masks (so with distribution to the population) for each time you leave the house in order to have a visual reminder of the need to follow recommendations, while also decreasing contamination.

Many advocated for free distribution to the entire population (including gloves and sanitizer if possible), or access at affordable prices.

Ensure that masks are available for everyone, even if their usefulness remains controversial.

#### Government Messaging

Although many participants considered the government messages and response adequate and expressed gratitude, some asked for more transparency and precise messages, centralized information, and specific information for groups at risk.

Give clear and comprehensible recommendations. Avoid jargon.

#### Lockdown and Lockdown Exit Plans

A considerable number of people favored slower easing of restrictions, or even an extension of lockdown with stricter measures, particularly for vulnerable people.

Do not rush deconfinement, it’s too early and I am at risk, it scares me…

Enforce complete confinement during at least two weeks.

Further, some called for tighter policing.

Be even more firm (fines? More insistent?) about hand washing and especially social distancing.

1. More surveillance and fines that are a stronger deterrent, 2. Systematic surveillance of rules in businesses.

A number of people recommended postponing the opening of schools and day cares, and making the return to school optional.

Wait longer before reopening schools, because with the recent reopening of stores and other places, there will be a resurgence in cases and if children return to school, it’s grandparents who will care for them after school because parents will be back at work.

#### Testing

Large-scale deployment of COVID-19 testing was a recurring theme.

Begin testing everyone with symptoms and not just those who are at risk or are medical personnel.

Further, the use of serological tests, the tracing of chains of transmission, and the isolation of positive cases were also mentioned repeatedly.

Impose electronic surveillance with tracking (smartphones) for all citizens during the pandemic.

## Discussion

In this cross-sectional, electronic survey distributed by social media, knowledge and self-reported adherence were high in this sample from Switzerland. Despite high overall satisfaction, free-text answers revealed a desire for greater availability of protective equipment, clearer and more coherent communication by the government, and large-scale testing for the virus. People in isolation and those with lower health literacy reported greater concerns about the pandemic, associations that persisted after controlling for demographic features.

While several larger survey studies on the same subject matter are underway in Europe [9], few studies with quantitative data about individual responses are available for comparison. A Chinese survey also showed high knowledge scores (10.8/12) and high confidence that COVID-19 would be controlled (91%) [10]. In an online survey by the Swiss public broadcasting corporation, 48% of respondents reported major concerns about COVID-19 and 49% felt the government response was too slow [11]. Satisfaction with the government response was higher in a second survey on April 8, 2020 [12]. Support for government restrictions was higher in Italian- and French-speaking areas, which had a higher density of cases, than in German-speaking areas of Switzerland. We did not find data regarding barriers and enablers of adherence to COVID-19 recommendations or data to be stratified by health literacy. Several other surveys conducted in the United States [5], United Kingdom [5], India [6], Egypt [13], and Malaysia [14] have shown adequate overall knowledge about COVID-19 among respondents. However, specific misconceptions and variations in knowledge according to age, income level, educational attainment, race, and ethnicity have been identified. A US national survey of 5198 people suggested that the largest differences in COVID-19–related knowledge and behaviors were associated with race/ethnicity, sex, and age [15]. African American participants, men, and people younger than 55 years were less likely to know how the disease spread, were less likely to know the symptoms, and left the home more often [15].

In our sample, self-reported adherence was high. A study of citizens’ adherence to COVID-19 mitigation recommendations in 3 countries (United States, Kuwait, and South Korea) suggested that in all 3 countries, government response efforts and business reopening agreements, as well as the intensity of information source use, social media use, and knowledge about COVID-19, all influenced either self-adherence or perception of the adherence of others [16].

The finding that those who had been in self-isolation and had lower health literacy were more worried about COVID-19 has important implications. Social isolation among older adults contributes to not only greater risk of depression and anxiety, but also cardiovascular, autoimmune, and neurological disease [17]. Thus, great care must be taken to help this group. Further, there is well-founded concern that marginalized groups may be disproportionately affected by government measures and the disease itself [18]. Information about COVID-19 in the media is often sensationalized and contradictory, making it challenging for those with lower health literacy to effectively assess risk. As far as could be determined, few studies have so far explored health literacy in the context of COVID-19–related knowledge and perceptions. One study of 630 US adults with chronic conditions found that, contrary to our sample, people who had lower health literacy were less likely to be worried about COVID-19 and to believe they would become infected [19]. Those with lower health literacy also reported greater confidence in the government response. A cross-sectional study of COVID-19–related health literacy was conducted among 1037 German people aged 16 and older [20]. The results highlighted that while the overall health literacy level was high, nearly half of respondents (47.8%) reported difficulties judging whether or not to trust media information on COVID-19. As expected, confusion about COVID-19–related information was significantly higher among respondents with lower health literacy. Accessible information, that adheres to widely accepted plain language principles, and is adapted to those who have lower health literacy and might feel most vulnerable, seems warranted.

Strengths of this survey included rapid data collection prior to easing restrictions, giving a first assessment of citizen responses to restrictions in Switzerland. The primary limitations were those inherent in cross-sectional, open, online survey samples. Moreover, self-reporting may be subject to desirability biases. We could not determine temporality between factors such as fear of the virus and self-isolation. Our sample had a higher proportion of women (76% versus 51%) and university graduates (65% versus 45%) than the Vaud population. Although rates of survey completion were high, we do not know our exact response rate. Finally, because of the novel subject matter, we could not use previously validated survey questions.

In conclusion, knowledge and self-reported adherence were high in an online sample from the Canton of Vaud. Levels of worry about COVID-19 were generally high, particularly among those in isolation and with lower health literacy. Future research should aim to better understand the concerns and needs of these groups and envision target support measures. Special effort will be needed to limit not only the direct health effects of COVID-19, but also psychological distress created by government restrictions as the pandemic continues to evolve.

## Appendix

Multimedia Appendix 1 English-language version of questionnaire.
