# Supplementary material for: Citizen Responses to Government Restrictions in Switzerland During the COVID-19 Pandemic: Cross-Sectional Survey
Source: JMIR Form Res. 2020 Dec 3;4(12):e20871. doi: 10.2196/20871 (PMC7717891; doi:10.2196/20871)
Supplement: Multimedia Appendix 1 [file formative_v4i12e20871_app1.pdf]

1. What is your age? ___ years

2. What is your sex? Male / Female / Other

3. How many people, including yourself, live in your household? ____________

4. In which Canton do you live? ____________

5. What is your highest level of education?
   - o Compulsory education or less
   - o Apprenticeship
   - o High school
   - o Professional school or university
   - o I don’t know

6. How confident are you filling out forms about your health by yourself (ex: questionnaire for new patients with a new doctor)?
   - o Compulsory education or less
   - o Apprenticeship
   - o High school
   - o Professional school or university

7. Have you been tested for the novel Coronavirus (COVID-19)?
   - o Yes, tested positive
   - o Yes, tested negative
   - o Yes, awaiting the result
   - o No
   - o I don’t know

8. Do you currently have the following symptoms: (check all that apply)
   - o Fever (≥38 C, feel feverish, or chills)
   - o Cough
   - o Difficulty breathing
   - o Other: Please specify
   - o No symptoms, I feel well
   - o I don’t know

Information about the novel coronavirus (SARS CoV-19)

9. What are your three primary sources of information to stay up to date with recommendations for COVID-19? (Check up to 3 responses)
   - o Government website or announcements
   - o Print or online news sources
   - o Information from television or radio

- Online blogs or other informal sources
- Social media (Facebook, Instagram, WhatsApp, Twitter, etc.)
- My employer
- My family and friends
- Government telephone hotline
- My healthcare providers or websites of hospitals or clinics
- I have not been staying up to date about the new coronavirus (COVID-19)
- Other: please specify

10. How worried, overall, would you say you are about the new coronavirus (SARS-CoV-19)? (1-10, with 1 not worried at all, 10 extremely worried)

11. Which of the following are recommended measures from the federal government to slow spread of the novel Coronavirus (COVID-19)? (check all that apply)
    - Wash my hands frequently for 20 seconds at a time (true)
    - Maximize time spent outdoors each day (false)
    - Carefully wash all fruits and vegetables (false)
    - Maintain 2 meters distance from others (true)
    - In case of fever or cough, stay home for 10 days and 48 hours without symptoms (true)
    - Stop using public transportation (false)
    - Cancel all public events (true)
    - Avoid mixing generations in a family (ex: grandparents keeping children) (true)
    - Work remotely if you can (true)

Changes to your routine because of the novel coronavirus (COVID-19)

12. Have you had to make any of these specific changes due to the novel Coronavirus (COVID-19) restrictions? (check all that apply)
    - Cancelled travel plans
    - Cancelled cultural event, sporting event, etc
    - Missed days of work
    - Worked remotely
    - Avoided physical contact with others (ex: shaking hands)
    - Cancelled private family or social engagements
    - Imposed self-isolation or self-quarantine, did not leave my home for days at a time
    - Have not had to make any changes
    - Other: please specify

13. What measures have you taken to protect your family and close friends?
    - I've provided them with information
    - I've helped them limit their exposure to many people
    - I've avoided visiting them
    - I avoided them when I had symptoms (ex: cough, fever, etc.)

- I’ve provided food and other essential products
- Does not apply to my situation
- Other: Please specify

14. What has helped you follow these measures? (Check all that apply)
    - Help from my family or friends
    - Help from my employer
    - Reimbursement from insurance or other sources (ex: reimbursed travel reservations)
    - Information from online sources
    - Information from friends or family
    - Information from government sources
    - Made changes to my habits or found tips and tricks to make it easier to follow recommendations
    - I followed the example of others
    - I haven’t followed the recommendations
    - Others: please specify

15. What has prevented, even occasionally, you from following these measures? (Check all that apply)
    - I don’t think the measures are useful to prevent spread
    - I don’t think the virus is dangerous for me or my family
    - Difficult to change my habits
    - Worry about offending others (ex: not shaking hands when offered)
    - Worry about missing time at work or disappointing my employer
    - Need to leave the house for essential products (ex: getting food)
    - Family obligations (ex: need to care for children or elderly)
    - I worry about my health status (ex: need to consult doctor)
    - I don't remember or understand the recommendations
    - I don’t have the means (ex: live in shared space)
    - I systematically follow the recommendations without any problems
    - Others: please specify

Following government recommendations

16. Overall, to what extent have *you yourself* adhered to federal recommendations, such as washing hands, maintaining 2 meters distance from others, avoiding touching your face, etc?
    (1 not at all - 10 in all situations)

17. Overall, to what extent have you *observed others* adhering to federal recommendations, such as washing hands, maintaining 2 meters distance from others, avoiding touching your face, etc?
    (1 not at all - 10 in all situations)

18. Have you intervened to encourage other individuals to follow these measures?

- Yes
- No
- I don’t know

19. Do you feel the government recommendations to limit coronavirus (COVID-19) to date have been:
(1 not at all enough   5 about right   10 much too restrictive)

18. What else could the government do to help individual citizens limit the spread of coronavirus (COVID-19)?

____

____

Automatic time stamp:___________
